# Peroxisomes and sexual development in fungi

**DOI:** 10.3389/fphys.2013.00244

**Published:** 2013-09-06

**Authors:** Leonardo Peraza-Reyes, Véronique Berteaux-Lecellier

**Affiliations:** ^1^CNRS, Institut de Génétique et Microbiologie, University Paris-Sud, UMR8621Orsay, France; ^2^USR3278 CRIOBE CNRS-EPHE, CRIOBEMoorea, Polynésie Française; ^3^Labex “CORAIL”, USR 3278 CNRS-EPHE, Centre de Recherche Insulaire et Observatoire de l'Environnement (CRIOBE)Moorea, Polynésie Française

**Keywords:** peroxisomes, peroxins, fungi, sexual development, meiosis, organelle biogenesis, cell differentiation

## Abstract

Peroxisomes are versatile and dynamic organelles that are essential for the development of most eukaryotic organisms. In fungi, many developmental processes, such as sexual development, require the activity of peroxisomes. Sexual reproduction in fungi involves the formation of meiotic-derived sexual spores, often takes place inside multicellular fruiting bodies and requires precise coordination between the differentiation of multiple cell types and the progression of karyogamy and meiosis. Different peroxisomal functions contribute to the orchestration of this complex developmental process. Peroxisomes are required to sustain the formation of fruiting bodies and the maturation and germination of sexual spores. They facilitate the mobilization of reserve compounds via fatty acid β-oxidation and the glyoxylate cycle, allowing the generation of energy and biosynthetic precursors. Additionally, peroxisomes are implicated in the progression of meiotic development. During meiotic development in *Podospora anserina*, there is a precise modulation of peroxisome assembly and dynamics. This modulation includes changes in peroxisome size, number and localization, and involves a differential activity of the protein-machinery that drives the import of proteins into peroxisomes. Furthermore, karyogamy, entry into meiosis and sorting of meiotic-derived nuclei into sexual spores all require the activity of peroxisomes. These processes rely on different peroxisomal functions and likely depend on different pathways for peroxisome assembly. Indeed, emerging studies support the existence of distinct import channels for peroxisomal proteins that contribute to different developmental stages.

## Introduction

Peroxisomes are single membrane-bound organelles that are highly dynamic and versatile. They are present in most eukaryotic organisms and involved in a number of essential metabolic pathways. Peroxisomes have an important role in lipid metabolism (Wanders et al., 2010) and are implicated in the homeostasis of reactive oxygen species (ROS) (Fransen et al., 2012). Ubiquitous metabolic pathways, like the β-oxidation of fatty acids, reside in peroxisomes throughout eukaryotes (Poirier et al., 2006). In addition, peroxisomes perform many diverse metabolic activities. Some of these activities have a relatively broad distribution among eukaryotes, like the glyoxylate cycle in plants, fungi and some protists (Huang et al., 2004; Kunze et al., 2006). Others are highly specific, like penicillin biosynthesis in the fungus *Penicillium chrysogenum* (Bartoszewska et al., 2011).

Peroxisomes are now recognized as important signaling organelles. Signaling molecules are generated by metabolic process in peroxisomes (Nyathi and Baker, 2006; Joo et al., 2010; Del Rio, 2011), which can modulate the activity of key signaling proteins (Li et al., 2000). Signaling proteins are also targeted directly into peroxisomes, where they integrate external signals to trigger specific cell developmental responses (Szoor et al., 2010). Furthermore, peroxisomes function as a scaffold for the assembly of specific macromolecular signaling complexes, which participate in the orchestration of complex signaling networks (Dixit et al., 2010; Horner et al., 2011).

Fungi provide a notable example of the functional versatility of peroxisomes. In addition to the ubiquitous roles shared with most eukaryotes, peroxisomes in fungi participate in metabolic pathways like methanol assimilation (Van Der Klei et al., 2006), biotin (Tanabe et al., 2011) and siderophore biosynthesis (Grundlinger et al., 2013). Moreover, several core glycolytic enzymes localize to peroxisomes in fungi (Freitag et al., 2012), a property until recently believed to be restricted to some Euglenozoa protists (Gualdron-Lopez et al., 2012). In addition, the fungal peroxisome is implicated in secondary metabolism and participates in the formation of metabolites like β-lactam antibiotics (penicillins, cephalosporin) and mycotoxins (AK-toxin, paxilline, aflatoxins) (Bartoszewska et al., 2011; Martin et al., 2012). Remarkably, peroxisomes impact the fungal cell dynamics by functions beyond their metabolic activity. For example, ascomycete fungi possess a specialized type of peroxisome, the Woronin body, which serve as plugs for septal pores that interconnect hyphal cell compartments (Jedd, 2011). Also, a subclass of peroxisomes has been implicated in the organization of the microtubule cytoskeleton in *Emericella nidulans* (anamorph: *Aspergillus nidulans*) (Zekert et al., 2010).

In agreement with their functional versatility, peroxisomes participate in diverse developmental processes, such as gametophyte recognition during fertilization in *Arabidopsis thaliana* (Boisson-Dernier et al., 2008) and the host-related morphogenic transitions of *Trypanosoma brucei* (Szoor et al., 2010). In animals, developmental processes like spermatogenesis (Chen et al., 2010; Nakayama et al., 2011; Baes and Van Veldhoven, 2012) and nervous system development (Faust et al., 2005; Baes and Van Veldhoven, 2006, 2012; Mast et al., 2011; Nakayama et al., 2011) require peroxisome activity, and their deficiencies cause severe and highly complex diseases in humans (Waterham and Ebberink, 2012). Fungi have provided numerous examples of developmental processes that depend on peroxisomes (reviewed in Peraza-Reyes et al., 2010). Different developmental events underlying the formation of asexual spores rely on peroxisomes. They also participate in developmental processes that define some fungal lifestyles, including the differentiation of infective structures, appresoria, in phytopathogenic fungi, in the yeast-mycelial transition of some dimorphic fungi, and in the development of nematode-trap cells by nematophagous fungi. The focal point of this review is the role of peroxisomes in the developmental process of fungal sexual reproduction.

## Peroxisome biogenesis, an overview

Peroxisome formation is mediated by conserved proteins known as peroxins, which are denoted by the Pex acronym. Peroxisomes can multiply by growth and division from pre-existing peroxisomes or be formed by budding from specific domains of the endoplasmic reticulum (ER) (Dimitrov et al., 2013). Formation of peroxisomal membranes and insertion of proteins into peroxisome membranes depend on the peroxins Pex3 and Pex19. On the other hand, the import of proteins into the luminal space of peroxisomes relies on a second group of peroxins, which are mostly peroxisome-membrane associated proteins (Liu et al., 2012; Pieuchot and Jedd, 2012; Theodoulou et al., 2013). Elimination of Pex3 and Pex19 abrogates peroxisome formation. In contrast, deletion of the peroxins that mediate the import of peroxisome matrix proteins results in peroxisome remnants devoid of luminal proteins.

Two highly conserved import pathways drive the peroxisome matrix protein import. These pathways are defined by the import receptors Pex5 and Pex7. These peroxins recognize the peroxisome proteins in the cytosol, by means of their peroxisome targeting sequences (PTS1 and PTS2, respectively), and mediate their import into the organelle. The activity of the receptor Pex7 depends on accessory proteins known as PTS2-coreceptors (Schliebs and Kunau, 2006). In plants, animals and probably some fungi, Pex5 provides the PTS2-coreceptor activity. In contrast, most studied fungi harbor additional specific peroxins to fulfill this task, like the yeast paralogous Pex18 and Pex21, and their filamentous-fungi functional equivalent Pex20.

Both import pathways converge at a peroxisome membrane-associated complex known as the importomer. This complex consists of docking and RING-finger subcomplexes and facilitates the translocation of proteins across the peroxisome membrane. Interestingly, the import receptor Pex5 itself, along with a docking-complex peroxin Pex14 forms a transient and highly dynamic channel, which likely constitutes the site of protein translocation across the peroxisomal membrane (Meinecke et al., 2010). Import receptors and their cargos are imported to peroxisomes, and after releasing their cargo proteins in the peroxisome lumen, import receptors are translocated back to the cytosol. This process is mediated by the peroxisomal receptor export machinery, or exportomer (Platta et al., 2013), and allows the receptors to be used in subsequent rounds of import. Failure to export the receptors results in a blockage of the import process.

## Sexual development in fungi

**Figure 1:**
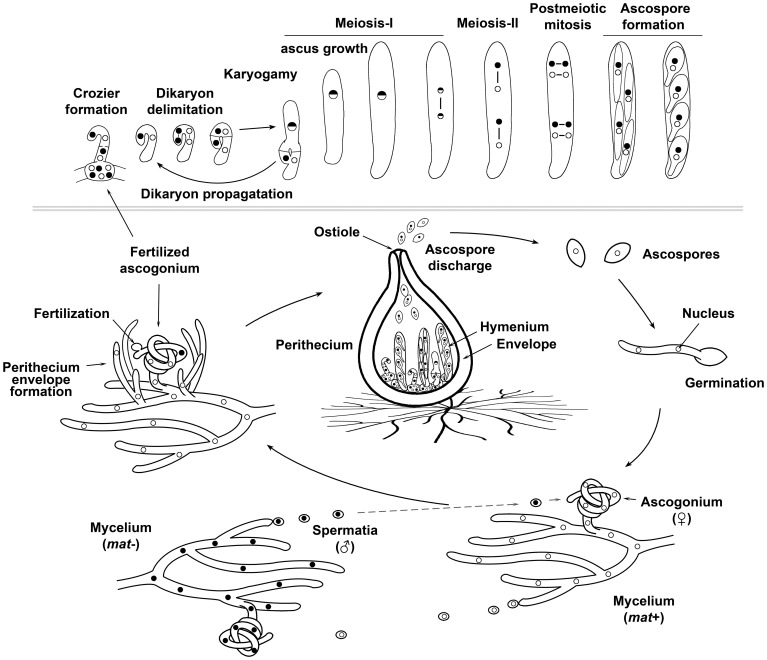
**The sexual life cycle of a model filamentous ascomycete fungus**. Sexual reproduction of most filamentous Ascomycetes takes place inside of multicellular fruiting bodies, which consist on sexual tissues (the hymenium) surrounded by a protective envelope. The hymenium is derived from mating and after karyogamy and meiosis produces sexual spores (ascospores), whereas the envelope tissues originate from maternal vegetative hyphae and are sterile. In a number of Ascomycetes, pyriform-shaped sexual fructifications are known as perithecia (singular perithecium). The sexual life cycle of filamentous Ascomycetes initiates by the differentiation of female organs (ascogonia), which originate as curved branches arising from vegetative hyphal cells. Ascogonia then become surrounded by aggregated hyphae, which eventually develop the perithecial envelope. Ascogonia can be fertilized by hyphae or asexual spores, but some fungi differentiate specialized cells that act as male gametes (spermatia). In heterothallic (self-sterile) fungi, fertilization only takes place between reproductive structures that differ genetically at their mating type (denoted in the figure as *mat*+ and *mat*− and illustrated by nuclei with different shading). After fertilization, the male gametic nucleus is delivered into the ascogonium, which contains the female gametic nuclei. This results in the formation of the hymenium. The upper inset illustrates the development of the hymenium from the dikaryotic stage to ascospore formation (from left to right): the two gametic nuclei (of opposite mating type in heterothallic fungi) are isolated in pairs in specialized hook-shaped cells called croziers. After coordinated mitoses (lines linking nuclei represent spindles) and septa formation, three cells are formed in each crozier: an upper binucleated and two flanking uninucleated cells. The two uninucleated cells fuse to produce a new dikaryotic crozier, which propagates the dikaryotic stage, whereas the upper dikaryotic cell undergoes karyogamy and develops into an ascus (the meiocyte). Meiosis takes place in this upper cell. Finally, the haploid nuclear products issued from meiosis are packaged into ascospores. In the figure, which illustrates the development of *P. anserina*, the eight nuclei issued from a post-meiotic mitosis are enclosed two by two into ascospores resulting in asci with four binucleated ascospores. Ascospores maturate inside the original mother ascus, from which they are ultimately forcibly ejected out.

Sexual reproduction in fungi exhibits a great diversity of reproductive strategies and mating systems (for review Casselton and Feldbrugge, 2010; Debuchy et al., 2010; Ni et al., 2011). The sexual life cycle of a model ascomycete fungus is shown in Figure 1. This process involves the alternation of haploid and diploid phases, which is sustained by the succession of karyogamy and meiosis, and provides the potential for genetic exchange. In general terms, sexual reproduction in fungi involves the differentiation of specialized mating structures that, after recognition of compatible partners, fuse to produce a zygote. In some fungi, karyogamy, and thus zygote formation, is concurrent to plasmogamy. However, in many fungal species, namely in Ascomycota and Basidiomycota (the Dikarya subkingdom), plasmogamy and karyogamy are separated in time by the propagation of a dikaryotic stage, which amplifies the number of karyogamies and meiocytes obtained from a unique fecundation event. Ultimately, meiosis takes place and for the majority of fungi the nuclear products of meiosis are packaged into sexual spores.

Sexual reproduction involves important changes in cellular architecture and often requires differentiation of multiple cell types. For many fungi, this process takes place inside of complex multicellular fruiting bodies. The formation of these structures occurs by a series of diverse cellular processes, including fusion, septation, branching, aggregation and adhesion of hyphae (Kues, 2000; Lord and Read, 2011). Therefore, sexual reproduction in fungi implicates a precise spatiotemporal coordination between various cell developmental events and progression through karyogamy and meiosis. Peroxisomes play an important role in the orchestration of these complex developmental processes.

## Peroxisomes are involved in the development of sexual reproductive structures

### Peroxisomes facilitate nutrient channeling to sustain the formation of sexual reproductive structures

Sexual reproduction in fungi frequently takes place when cells have exhausted the external nutrients and reach stationary phase. Under these circumstances, sexual development is mainly sustained by nutrients provided by the pre-existing vegetative cells from where the differentiated cells arise. The translocation of nutrients from vegetative hyphae is of particular importance for the formation of fruiting bodies, large and complex structures that in many fungi emerge from the substrate to grow into the air (Wosten and Wessels, 2006). The nutrients provided by the mycelium are derived largely from reserve compounds, such as carbohydrate and lipid reserves, or are generated by cell auto-assimilative processes, like autophagy (Bartoszewska and Kiel, 2011). Peroxisome metabolism can play an important role in channeling some of these nutrients, most notably the lipids.

**Figure 2:**
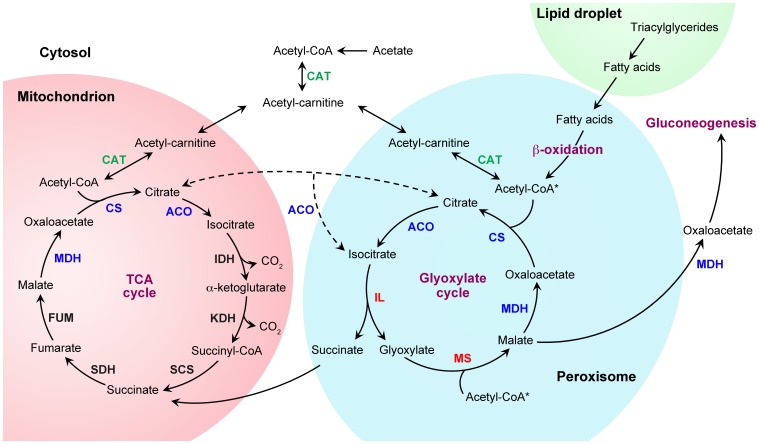
**Metabolic pathways for fatty acid and acetate utilization**. Triacylglycerides, which accumulate inside of lipid droplets, are converted to fatty acids and transferred to peroxisomes for catabolism. Fatty acids are converted to acetyl-coenzyme A (-CoA) by the fatty acid β-oxidation pathway. In ascomycete yeasts like *Saccharomyces cerevisiae*, the β-oxidation pathway is exclusively peroxisomal (Hiltunen et al., 2003; Shen and Burger, 2009). However, in most other fungi, this pathway occurs in peroxisomes and mitochondria (Maggio-Hall and Keller, 2004; Boisnard et al., 2009; Shen and Burger, 2009; Kretschmer et al., 2012a,b; Patkar et al., 2012) (only the peroxisomal pathway is illustrated). The anaplerotic glyoxylate cycle then allows the conversion of acetyl-CoA to four-carbon dicarboxylic acids by bypassing the decarboxylation reactions of the tricarboxylic acid (TCA) cycle (note that two reactions can feed acetyl-CoA into the glyoxylate cycle, asterisks). The glyoxylate shunt intermediates can replenish the TCA cycle or serve as precursors for gluconeogenesis (shuttling between compartments of some intermediates, such as malate and oxaloacetate, is not illustrated). The glyoxylate cycle consists of five enzymatic reactions, of which three are shared with the TCA cycle. Depending on the fungal lineage, these three enzymatic steps (blue font) can take place in peroxisomes, mitochondria or the cytosol (except for citrate synthase, for which no cytosolic form has been described so far). In contrast, the specific enzymes of the glyoxylate cycle, isocitrate lyase and malate synthase (red font), are typically peroxisomal (Kunze et al., 2006; Hynes, 2010; Strijbis and Distel, 2010). The most notable exception is *S. cerevisiae*, which harbors cytosolic isocitrate lyase (Taylor et al., 1996). Peroxisomal β-oxidation-produced acetyl-CoA can also be transferred to mitochondria for energy generation via TCA cycle. This transport depends on the interconversion of acetyl-CoA and acetyl-carnitine by carnitine acetyl-CoA transferases (green font). Acetyl-carnitine can then be imported into mitochondria by an acyl-carnitine carrier protein (Strijbis and Distel, 2010). Fatty acyl-CoA β-oxidation intermediates may also be transferred to mitochondria for further β-oxidation and acetyl-CoA generation within mitochondria (not shown). Cytosolic acetyl-CoA, which can be produced from C2 compounds, can also be transferred via acetyl-carnitine to mitochondria and peroxisomes for metabolism via the TCA and glyoxylate cycles. Only relevant enzymes are shown, abbreviations are as follows: ACO, aconitase; CAT, carnitine acetyltransferase; CS, citrate synthase; FUM, fumarase; IDH, isocitrate dehydrogenase; IL, isocitrate lyase; KDH, α-ketoglutarate dehydrogenase; MDH, malate dehydrogenase; MS, malate synthase; SCS, succinyl CoA synthetase; SDH, succinate dehydrogenase.

Storage lipids, which are primarily triacylglycerides, accumulate inside of lipid droplets (also known as lipid bodies) (Murphy, 2012). These organelles are derived from the ER and exhibit a close association with mitochondria and peroxisomes, which degrade fatty acids released from triglycerides to produce energy and biosynthetic metabolites via fatty acid β-oxidation and the glyoxylate cycle (refer Figure 2 for an outline on these metabolic pathways). Interestingly, association of peroxisomes with lipid droplets is so intimate that peroxisomes even invade the core of the lipid droplets by protrusive structures, pexopodia, that may facilitate the transfer of lipids into peroxisomes (Binns et al., 2006).

Early research on the regulation of intermediate metabolism in fungi revealed that the sexual fruiting bodies of basidiomycetes like *Coprinopsis cinerea* and *Schizophyllum commune* contain very low levels of isocitrate lyase activity, a glyoxylate cycle specific enzyme. In contrast glycolysis, together with the tricarboxylic acid (TCA) cycle, represent the major pathways for carbohydrate metabolism (Cotter et al., 1970; Schwalb, 1974; Moore and Ewaze, 1976). Two main scenarios could account for these observations. First, the glyoxylate cycle, as well as the β-oxidation of fatty acids, could be dispensable for fruiting body development. Alternatively, fatty acid β-oxidation and the glyoxylate shunt could take place in the mycelium that produces the fruiting bodies, and the glyoxylate cycle-derived intermediates could be transported into the developing fructifications. A number of lines of evidence now indicate that both of these scenarios occur in fungi.

In the brown-rot basidiomycete *Fomitopsis palustris*, comparison of the enzymatic activities of the TCA and glyoxylate cycles suggests that the glyoxylate bypass has no prominent role within fruiting bodies. Furthermore, even when both specific enzymes of the glyoxylate cycle, isocitrate lyase and malate synthase, exhibit high enzymatic activity in young mycelium; they are significantly down-regulated much before fruiting body formation. Thus, no correlation is observed in this fungus between the glyoxylate cycle activity and the developmental stages where sexual fruiting bodies are produced (Yoon et al., 2002a).

In contrast, in the white-rot basidiomycete *Flammulina velutipes* (the Enokitake mushroom), the activity of malate synthase and isocitrate lyase are significantly higher in the mycelial mats producing fruit bodies compared to equivalent mycelia than do not produce fructifications. In addition, the mycelial activity of malate synthase increases during fruiting-body development (Yoon et al., 2002b). This suggests an important role for the glyoxylate cycle in the vegetative cells supporting fruiting-body formation in *F. velutipes*.

The participation of the glyoxylate cycle in the formation of sexual reproductive structures has been demonstrated in the cereal pathogen *Gibberella zeae* (anamorph: *Fusarium graminearum*). In this ascomycete, the transcript levels of the isocitrate lyase-encoding gene, *ICL1*, are high in mycelia before sexual development, and become negligible in late stages of fruiting-body (perithecium) development. In this fungus, the repression of *ICL1* occurs after perithecia induction. Furthermore, deletion of *ICL1* results in a severe reduction in the number of produced perithecia (Lee et al., 2009). These data indicate that in this fungus the glyoxylate cycle is, in fact, required for fruiting body formation, and suggest a channeling of glyoxylate cycle-derived intermediates from the vegetative mycelium into developing perithecia.

The glyoxylate shunt in *G. zeae* is required for fatty acid utilization (Lee et al., 2009); thus, the requirement of the glyoxylate cycle for perithecia formation may, indeed, be related to the mobilization of storage lipids. A critical event for reserve lipid utilization is the shuttling between peroxisomes and mitochondria of β-oxidation-produced acetyl-coenzyme A (-CoA) to allow the production of energy via the TCA cycle. This transport depends on the carnitine acetyltransferase-mediated acetyl-carnitine shuttle (Figure 2) (Strijbis and Distel, 2010). *G. zeae* mutants defective for a peroxisomal/mitochondrial carnitine acetyltransferase (CAT) produce fewer perithecia than a wild-type strain (Son et al., 2012). This finding is consistent with a role for the fatty acid β-oxidation pathway in sustaining fruiting-body formation. Interestingly, in these mutants, perithecia are produced only by some sectors of the mycelial colonies. This suggests that nutrients may be channeled into specific sectors of the mycelium to sustain the formation of limited numbers of fructifications when reserve nutrient mobilization is inefficient.

A genome-wide analysis of gene expression during sexual development in *G. zeae* revealed an expression pattern for the triacylgliceride metabolism genes that is consistent with lipid accumulation before sexual reproduction and lipid oxidation early during fruiting-body development (Guenther et al., 2009). This expression pattern is characterized by an up-regulation in mycelium preceding sexual development of the majority of lipid biosynthesis genes, which are subsequently repressed when perithecia start forming. This down-regulation of lipid biosynthesis genes occurs concomitantly with an up-regulation of lipid oxidation genes, which include the genes for the β-oxidation of fatty acids. Most peroxin-encoding genes followed the expression pattern of the lipid oxidation genes, suggesting an involvement of peroxisomes in this process (Guenther et al., 2009). Guenther and coworkers also observed high levels of lipid droplet accumulation in hyphae competent for sexual reproduction. This accumulation of lipids is notable in the specialized hyphae from where perithecia emerge, as well as in perithecium initials themselves, which are filled with densely packed lipid droplets (Guenther et al., 2009). Altogether, these data indicate that reserve lipids accumulate in the vegetative phase preceding sexual development and are later oxidized by β-oxidation to produce, along with the glyoxylate cycle, energy and biosynthetic intermediates to support sexual fruiting body formation in *G. zeae*. Consistent with participation of peroxisomes in these processes, perithecia formation is also severely reduced in mutant strains defective for peroxisome formation (Min et al., 2012).

### Peroxisomes may facilitate nutrient channeling from asexual to sexual differentiated multicellular structures

A nice example of the diversity of developmental systems leading the formation of reproductive structures in fungi is observed in the ascomycete *Sclerotinia sclerotiorum*. In this devastating plant pathogen, sexual fruiting bodies (referred to as apothecia) consist on large cup-shaped structures formed by a stipitate disc where the sexual tissues (the so-called hymenium) are embedded. In this fungus, apothecia do not arise from the mycelium, but from a second differentiated multicellular structure known as the sclerotium. Sclerotia are asexual resistance structures that consist of densely packed hyphal aggregates coated by a rind of highly melanized hyphae (Erental et al., 2008).

In *S. sclerotiorum*, differentiation of apothecia is affected by disruption of *pth2*, which encodes a peroxisomal CAT. The elimination of this protein results in fructifications with short stipes and disks that do not fully expand (Liberti et al., 2013). Since *pth2* is also required for fatty acid β-oxidation, it is possible that fatty acid utilization is also required for apothecia formation. Moreover, since the resources supporting sexual fruiting-body formation arise from sclerotia and not from mycelia, peroxisomes may also be important for nutrient channeling between distinct multicellular fungal structures. However, the identity of the sclerotial reserve nutrients supporting apothecium formation is not well-understood. Whereas a high sclerotium lipid content and the presence of hyphae with a rich content in lipid bodies has been documented (Calonge, 1970; Kosasih and Willetts, 1975), it has also been suggested that sclerotium carbohydrates rather than lipids sustain apothecium formation (Weete et al., 1970; Coley-Smith and Cooke, 1971). Consequently, it has been postulated that the role of Pth2 in apothecium formation could be regulatory rather than nutritional (Liberti et al., 2013). Further research should provide relevant information on this issue.

### Peroxisomes may also facilitate nutrient channeling between different cell types within fructifications: evidence from the truffles

In the ecto-mycorrhizal fungus *Tuber borchii* (the whitish truffle), the transcripts for isocitrate lyase and malate synthase are much more abundant in fruiting bodies at different stages of maturation than in the vegetative mycelium (Lacourt et al., 2002; Abba et al., 2007). Likewise, an enhanced accumulation of the transcripts coding for glyoxylate cycle enzymes is observed in the fruiting bodies of *Tuber melanosporum* (the Perigord black truffle) (Ceccaroli et al., 2011). This suggests that in these Ascomycetes the glyoxylate cycle is required for later stages of fruiting-body development, which could include fruiting-body enlargement, meiotic development and ascospore (the meiotic-derived sexual spore of Ascomycetes) differentiation. Interestingly, in *T. borchii* lipid droplets are abundant in the vegetative cells in young fructifications. The lipid droplets are not observed in these cells after fruiting-body maturation but do accumulate in mature ascospores (Abba et al., 2007). This suggests that a relocation of metabolic resources also takes place between different cell types of the fruiting bodies along their development in some fungi.

### Lifestyle-related implication for peroxisomes in fruiting body development, an example from phytopathogenic fungi

Importantly, fungi discussed above differ in their phylogenetic origin and in their lifestyle and reproductive systems. Thus, different requirements for the fatty acid β-oxidation and glyoxylate cycle during development may reflect diverse ways evolved in fungi with different lifestyles to sustain fruiting-body development. This is also evident when we consider the correlation between sexual development and the plant infection cycle in *G. zeae*. During the infection of wheat, formation of perithecium initials by the fungus begins when the fungus-colonized plant tissues begin to senesce. The perithecium initials function as overwintering structures on plant debris, and resume their development to form perithecia and ultimately produce ascospores that infect new plants at permissive temperatures (Trail, 2009). Notably, the transcriptional regulation pattern of the genes involved in lipid biosynthesis and oxidation observed during sexual development in culture also occurs during the equivalent developmental stages *in planta* (Guenther et al., 2009). This indicates that lipid reserves that accumulate in hyphae and perithecium-initials during plant colonization are used for overwintering and later oxidized to support perithecium development upon favorable climatic conditions.

On the other hand, the transcriptional regulation pattern of the lipid metabolism genes during development in *G. zeae* is similar, but not completely alike, in the related cereal pathogen *Gibberella moniliformis* (anamorph: *Fusarium verticillioides*). In contrast to *G. zeae*, the induction of lipid biosynthesis genes prior sexual development in *G. moniliformis* is moderate, and this fungus harbors elevated transcript levels of lipid oxidation genes even before sexual development (Sikhakolli et al., 2012). These differences could be interpreted in terms of their reproductive system. While sexual development seems to be critical for the homothallic (self-fertile) *G. zeae* infection cycle, the heterothallic (self-sterile) *G. moniliformis* seems to be sexually less prolific. The constantly high transcript levels of lipid oxidation genes in *G. moniliformis* could indicate that these genes also participate in the asexual spore formation process, which precedes sexual development and is more profuse than in *G. zeae* (Sikhakolli et al., 2012).

### Additional roles for peroxisomes during fruiting body formation

The formation of sexual fruiting bodies in saprophytic ascomycetes like *Aspergillus nidulans, Podospora anserina* and *Neurospora crassa* is affected when peroxisome biogenesis is compromised (Bonnet et al., 2006; Managadze et al., 2007; Hynes et al., 2008). *A. nidulans* produce small fruiting bodies (cleistothecia) in homozygous crosses whenever the PTS1 peroxisome matrix protein import is impaired. This phenotype is not observed in *pexG* (*pex7*) mutants, where only the PTS2 import pathway is affected. In *A. nidulans*, isocitrate lyase import into peroxisomes depends on PexG and peroxisomal localization of malate synthase (a PTS1-containing protein) is not essential for a functional glyoxylate cycle (Hynes et al., 2008). Moreover, deletion of *acuJ*, which encodes a peroxisomal/mitochondrial CAT essential for acetate and fatty-acid utilization, has no detectable developmental defects (Hynes et al., 2011). These observations suggest that the deficiency in cleistothecia formation resulting from defective PTS1 peroxisome protein import is not caused by an impaired β-oxidation/glyoxylate cycle-dependent nutrient channeling. Thus, there may be additional functions for peroxisomes in fruiting body development in *A. nidulans*.

In *P. anserina*, deletion of the PTS1 receptor PEX5 results in a reduction in the size and number of perithecia produced by this fungus (Bonnet et al., 2006). This developmental phenotype is not produced by defects in β-oxidation of fatty acids (Boisnard et al., 2009). Thus, in *P. anserina* and *A. nidulans*, peroxisomes have additional roles during fruiting body development beyond the β-oxidation pathway. However, unlike *A. nidulans*, deletion of *P. anserina pex7*, which encodes the PTS2 receptor PEX7, partially suppresses the perithecium development phenotype observed upon deletion of *pex5* (Bonnet et al., 2006). This indicates that mislocalization of all proteins that undergo PEX5- and PEX7-dependent import into peroxisomes is less detrimental compared to mislocalization of peroxisomal matrix proteins that require PEX5 for import. Furthermore, the perithecium developmental phenotype of *P. anserina pex5* mutants displays a maternal effect: it is observed both in homozygous and heterozygous (to a wild-type strain) crosses whenever the *pex5* mutant acts as female partner (Bonnet et al., 2006). This indicates that peroxisomes are required for the development of maternally derived tissues. These tissues could be concerned with the formation of the perithecium envelope (see Figure 1), which in ascomycete fungi like *P. anserina* is exclusively of maternal origin (Debuchy et al., 2010).

In *N. crassa*, peroxisomes could be required at a very early stage of perithecium development, as mutant strains deficient for the docking-complex peroxin PEX14 are female sterile and do not produce perithecia (Managadze et al., 2007). Interestingly, no similar phenotype has been associated to other fungal mutations affecting peroxisome biogenesis, including *pex14* deletion in *P. anserina* (Peraza-Reyes et al., 2008). This may indicate fundamental differences in sexual determination even in closely related fungi.

## Peroxisomes are involved in the formation of signaling molecules that regulate sexual development

An additional function of peroxisomes during sexual reproduction stems from their participation in the formation of signaling molecules. In *A. nidulans*, the formation of sexual reproductive structures and asexual spores is affected by mutations that abolish PTS1 peroxisome import. These mutations also exacerbate the sexual development phenotype of a *veA1* mutant and result in very decreased cleistothecia formation (Hynes et al., 2008). The *veA1* mutation affects the velvet protein VeA, a regulatory protein that coordinates the balance between sexual and asexual development (Bayram and Braus, 2012). The development of this fungus is also coordinated by *psi* factors, fatty acid-derived oxylipin pheromones. *Psi* factors are secondary metabolites produced by hydroxylation of oleic, linoleic and linolenic acids. The ratio of *psi* factors determines the balance between asexual and sexual development (for review, Tsitsigiannis and Keller, 2007). In *A. nidulans*, oleic acid stimulates the production of cleistothecia and reduces asexual sporulation. Importantly, deletion of PexF (Pex6, an exportomer peroxin) inhibits oleate-stimulated cleistothecium formation. These observations suggest that the peroxisome metabolism affects the levels of oxylipin *psi* factors, which control the balance between sexual and asexual development in *A. nidulans* (Hynes et al., 2008).

The acyl-CoA-binding protein Acb1 of *Pichia pastoris* is another signaling molecule whose formation depends on peroxisomes. Acb1 is a conserved protein that is secreted and proteolytically processed to produce an extracellular signaling peptide. In the yeast *P. pastoris*, Acb1 secretion is induced by nitrogen starvation and is required for ascospore formation (Manjithaya et al., 2010). Interestingly, Acb1 secretion occurs by an unconventional pathway that relies on autophagosome-like vesicles and not on the classical ER/Golgi secretory system (for review, Rabouille et al., 2012). Importantly, Acb1 secretion requires peroxisome biogenesis and formation of medium-chain fatty acyl CoA inside peroxisomes. Thus, it has been postulated that Acb1 binding to medium-chain fatty acyl CoA produced within peroxisomes is critical for Acb1 secretion (Manjithaya et al., 2010). Intriguingly, however, mutants of *P. pastoris* defective for the importomer, which is required for Acb1 secretion (Manjithaya et al., 2010), do not display sexual cycle defects (Waterham et al., 1996). Therefore, the precise participation of peroxisomes during this process awaits further examination. Nonetheless, these observations provide an interesting example of how the peroxisome function can impact different cellular processes implicated in the formation of signaling molecules important for sexual development.

## Involvement of peroxisomes in mating, an example from the virulence-related sexual development of a plant pathogen

*Ustilago maydis* (the cuitlacoche or corn smut) is a basidiomycete plant pathogen that completely depends on the infection of its host (maize) to complete its sexual cycle (reviewed in Vollmeister et al., 2012). Haploid yeast-like cells of this fungus proliferate saprophytically by budding. However, in order to infect its host and to undergo sexual reproduction, the growth pattern must be switched to produce filamentous cells. This transition requires the pheromone-mediated recognition of mating partners, and results in the formation of conjugation hyphae. These cells undergo plasmogamy by fusing their tips and generate a dikaryotic hypha, which is the infectious cell type of *U. maydis*. The dikaryotic hyphae proliferate in the plant and ultimately differentiate into diploid teliospores (resting spores competent to undergo meiosis of some Basidiomycetes), which are produced inside of fungus-induced plant tumors (Vollmeister et al., 2012).

A third example of a signaling role for peroxisomes is observed in the virulence-related sexual cycle of *U. maydis*. This example also illustrates an interesting connection between peroxisomes in sexual-development and the infection cycle of a plant pathogen. Fatty acids, as well as their hydroxylated derivatives (which are components of the plant cuticle cutin), induce filamentation in *U. maydis* (Klose et al., 2004; Mendoza-Mendoza et al., 2009). Interestingly, the switch to filamentous growth induced by fatty acids depends on the β-oxidation pathway and, depending on the fatty acid, mitochondrial and/or peroxisomal pathways are required for this induction (Klose and Kronstad, 2006; Kretschmer et al., 2012a).

The ability to mate also depends on the β-oxidation of fatty acids. The mating ability of cells deficient for the peroxisomal β-oxidation is reduced in heterozygous crosses, and weak in homozygous crosses; whereas it is only slightly reduced in homozygous crosses of cells deficient for the mitochondrial β-oxidation (Kretschmer et al., 2012a). These observations indicate that the developmental switch resulting in hyphal growth in *U. maydis* is regulated by intermediate metabolites or fatty acid derivatives that are produced by the fatty acid β-oxidation. They also reveal a role for peroxisomes in the differentiation process upholding mating in *U. maydis*. Interestingly, proliferation of dikaryotic hyphae in the host tissues and the formation of plant tumors is also reduced by deficiencies in the β-oxidation pathway (Klose and Kronstad, 2006; Kretschmer et al., 2012a). Moreover, the differentiation of teliospores is significantly delayed when there are defects in peroxisomal β-oxidation (Klose and Kronstad, 2006). Whether this developmental delay results from the lower hyphae proliferative efficiency or from additional roles for β-oxidation in teliospore differentiation remains to be established. Nevertheless, altogether, these observations indicate that the fatty acid β-oxidation has a profound and complex impact on the virulence-related sexual development of *U. maydis*.

## Peroxisome involvement in meiotic development and sexual sporulation

### Peroxisome dynamics and assembly

#### Peroxisome dynamics is differentially regulated during meiotic development and sexual spore formation

The first report that peroxisome number can be highly variable between fungal cells was published nearly 30 years ago. Veenhuis and collaborators (1984) observed that during nematode infection the trap cells of the nematophagous fungus *Arthrobotrys oligospora* were filled with peroxisomes, while vegetative cells from where trap cells emerge were not. This observation indicated that the dynamics of peroxisomes is regulated during development. Peroxisome dynamics during sexual development has been studied in the yeast *Saccharomyces cerevisiae* and in the filamentous fungus *P. anserina*. The sexual cycle of *S. cerevisiae* is initiated when haploid cells of opposing mating types encounter each other and fuse to produce a zygote, which propagates asexually by budding. When diploid cells are exposed to nutritional limitations, they undergo meiosis and pack their resulting haploid nuclei into ascospores. The four resulting ascospores—the tetrad—are encased inside the original mother cell, the ascus [for a comprehensive review on *S. cerevisiae* sporulation see Neiman (2011)].

After mating, peroxisomes from each parental cell are transferred into the zygote, where no mixing of their contents is observed (Motley and Hettema, 2007). Then, during sporulation, peroxisomes are observed throughout the two meiotic divisions and they partition to the four cellular products of meiosis. Thus, peroxisomes are partitioned during sporulation. During this process, the number and distribution of peroxisomes are not significantly altered in *S. cerevisiae* (Gurvitz et al., 1998). Interestingly, the number, size and localization of peroxisomes is tightly regulated during the sexual development in filamentous Ascomycetes. In *P. anserina* (please refer to Figure 1 to appreciate the sexual development of this fungus), the sexual differentiated female organs (ascogonia) contain peroxisomes that are mainly round or elongated, and have an even distribution along the septated hyphae. This arrangement is similar to the one observed in vegetative hyphae from where ascogonia are formed (Peraza-Reyes et al., 2009). After fertilization, the dikaryotic crozier cells contain few peroxisomes (Berteaux-Lecellier et al., 1995; Peraza-Reyes et al., 2011), but their number increases importantly after karyogamy in the ascus (meiocyte).

After nuclear fusion, the diploid nucleus enters meiosis and the ascus elongates from 10 to over 150 microns (see Figure 1). Hundreds of peroxisomes can be observed in the young growing asci, a large portion of these peroxisomes is clustered at the tip of the cell. By the end of meiotic prophase-I, asci reach their final length and peroxisomes are more evenly distributed along the cell. The number of peroxisomes remains constant from metaphase-I to ascospore formation. However, peroxisomes proliferate during ascospore maturation (Berteaux-Lecellier et al., 1995; Bonnet et al., 2006; Peraza-Reyes et al., 2009, 2010). Altogether, these observations indicate that peroxisome dynamics is highly regulated during sexual development in filamentous fungi, implying a differential requirement for these organelles throughout sexual development progression. These observations also suggest that the location where peroxisomes act is important for the development of specific sexual cells.

#### Peroxisome assembly is also differentially regulated during meiotic development and sexual spore formation

**Figure 3:**
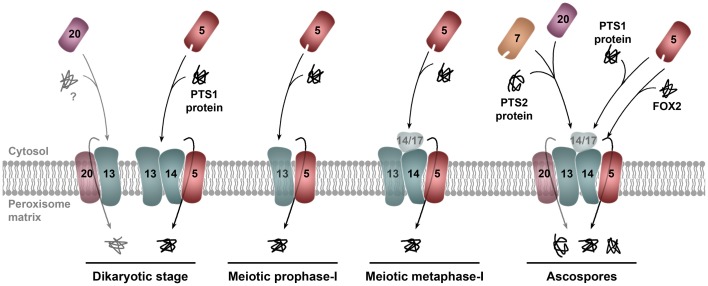
**Model for the regulation of the docking/translocation machinery for peroxisome matrix protein import during sexual development in *P. anserina***. The peroxins of the docking/translocation machinery are differentially required during sexual development in *P. anserina*. PEX14 (only the peroxin number is indicated) is required for peroxisome matrix protein import at the dikaryotic stage, in asci after the first meiotic metaphase and in ascospores; but not during the first meiotic prophase or in the early stages of ascospore differentiation (not depicted). PEX14/17 is involved in the import of matrix proteins in meiosis after metaphase-I, but it is not required for import in young differentiating ascospores (not illustrated). Upon ascospore maturation, PEX14/17 is required for the import of PTS1-containing proteins, but not for the PEX5-dependent import of proteins missing PTS1 signals, like the peroxisomal fatty acid β-oxidation multifunctional enzyme FOX2. The third docking protein, PEX13, is likely required for the activity of the docking/translocation machinery throughout meiotic development; however, absence of this protein blocks meiocyte formation. Thus, its function during meiotic development cannot be assessed (depicted with translucent shading). In addition to PEX13 (and unlike PEX5, PEX7, PEX14, or PEX14/17), PEX20 is also required for meiocyte formation, which suggests an alternative import pathway required at the dikatyoric stage for meiocyte formation. PEX20 could provide a pore-forming activity (illustrated by a translucent membrane location) additional to PEX5 for the translocation of proteins across the peroxisomal membrane (see text and Peraza-Reyes et al., 2011 for details).

Remarkably, the activity of the protein machinery that drives the import of proteins into peroxisomes is also tightly coupled with sexual development. A critical component of the protein translocation machinery for peroxisome matrix protein import is PEX14 (Azevedo and Schliebs, 2006; Liu et al., 2012). This protein is a conserved component of the docking complex and has been implicated in formation of the protein translocation channel in *S. cerevisiae* (Meinecke et al., 2010). PEX14 is required for peroxisome matrix protein import in vegetative hyphae of *P. anserina*. Interestingly, its requirement for import during the sexual cycle is cell specific (Peraza-Reyes et al., 2011). PEX14 is necessary for import in dikaryotic croziers, in asci after meiotic metaphase-I and in ascospores. However, it is not essential for the growing meiotic-prophase-I asci (Figure 3) or for early stages of ascospore differentiation. Thus, the translocation channel for protein import can be assembled in absence of PEX14 during specific stages of sexual development. This could indicate that the constitution of the import channel differs at distinct developmental stages. Alternatively, there is evidence that Pex5 constitutes the central core of the peroxisome translocation channel (Salomons et al., 2000; Kerssen et al., 2006; Meinecke et al., 2010) and Pex14 could facilitate its assembly. Therefore, it is possible that assembly of the translocation channel could be differentially regulated during sexual development. Under both scenarios, the differential requirements for PEX14 during sexual development may reflect differences in the functional state of the translocation machinery (Figure 3).

Meiotic prophase-I, the stage when recombination occurs, is a critical stage of sexual reproduction. The observation that peroxisome import takes place at this stage even in absence of PEX14 could indicate an important role for peroxisomes at this stage. Interestingly, a second *P. anserina* importomer peroxin is required at some but not all stages of sexual development. This peroxin, PEX14/17, is related to but not redundant with PEX14 (Peraza-Reyes et al., 2011). Furthermore, PEX14/17 and PEX14 are not required at the same stages of sexual development, and elimination of PEX14/17 affects the specificity of the import at certain developmental stages; notably, PEX14/17 is required in ascospores for the import of PTS1-containing proteins but not for the import of PEX5 cargos that lack PTS1 signals (Figure 3). Since neither PEX14 nor PEX14/17 is absolutely required for import at meiotic prophase-I and in young differentiating ascospores, additional importomer components should sustain the activity of the docking/translocation machinery at these stages. One such component could be the third docking-complex peroxin PEX13 (Figure 3). Interestingly, this protein is required for meiocyte formation *per se* (see below), which has precluded analyzing its role in import during meiotic development. Altogether, these observations indicate that the functional state of the importomer is regulated during sexual development in *P. anserina*, and suggest that an additional regulation of the peroxisome constitution and function during development is exerted by a differential modulation of the protein complex that selectively drives the import of proteins into the organelle.

### Peroxisomes are required for meiotic development

#### A role for peroxisomes in the initiation of meiotic development

Consistent with their differential dynamics and assembly regulation, peroxisomes are required for specific processes of meiotic development. Notably, one such process is the induction of meiotic development *per se*. In *P. anserina*, the RING-finger peroxin PEX2 was discovered as a protein required for the transition from the pre-karyogamy dikaryotic and mitotic phase to the karyogamy and meiotic phase of the life cycle (Simonet and Zickler, 1972, 1978; Berteaux-Lecellier et al., 1995).

**Figure 4:**
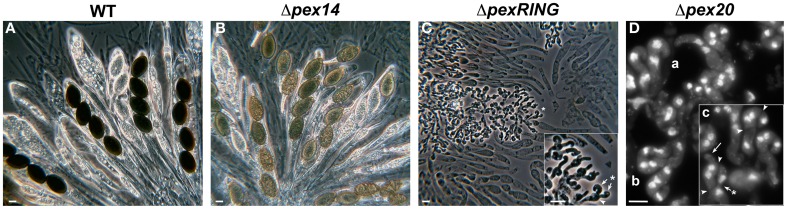
**Examples of sexual development defects produced by deficient peroxisome biogenesis in *P. anserina***. Sexual fruiting bodies of *P. anserina* wild-type **(A)** or Δ*pex14* **(B)** homozygous crosses contain asci with four ascospores. In absence of PEX14, ascospore pigmentation is deficient **(B)**. In contrast, perithecia from RING-finger-complex **(C**, here a Δ*pex2*Δ*pex10*Δ*pex12* strain**)** or Δ*pex20* **(D)** mutants contain only crozier-shaped dikaryotic cells. Image in **(D)** shows the nuclei distribution in these cells (DAPI staining). Lowercase letters indicate progressive developmental stages of crozier differentiation: **(a)** binucleated young croziers bending. **(b)** After simultaneous mitoses, each crozier contains four nuclei. **(c)** Septa formation (arrowheads) delimitates a dikaryotic upper cell from uninucleate lateral and basal cells. The uninucleated cells fuse (asterisk arrow) and the lateral-cell nucleus migrates into the basal compartment. Instead of undergoing karyogamy and meiosis as in the wild type, the nuclei from the upper dikaryotic crozier cell will be engaged in the formation a new crozier (arrow). This results in crozier “trees” where no asci are produced, as shown in **(C)**. Note how each original crozier (inset in **C**, arrowhead) produces two new croziers (arrows). Scale bars, **A–C**: 10 μm; **D**: 5 μm.

Mutant strains defective for *pex2* are sterile in homozygous crosses and their sexual development is blocked at the dikaryotic stage prior to formation of asci (meiocytes) and ascospores (Figure 4C, compare to A). In these mutants, the dikaryotic crozier cells differentiate normally (illustrated in Figure 4D for a *pex20* mutant, which exhibits the same developmental phenotype as *pex2* mutants, see below) and the coordinated mitoses that lead to dikaryon formation are synchronous with their spindle and spindle pole bodies (SPBs, the nuclear-embedded functional analogs of centrosomes) correctly formed (Simonet and Zickler, 1972). Like in a wild-type strain, these mitoses result in an upper dikaryotic cell flanked by two uninucleated cells, which will further fuse to form a new basal bi-nucleated cell. Normally, the upper dikaryotic cell develops into a meiocyte and the basal cell differentiates a new crozier, which perpetuates the dikaryotic stage (see Figure 1). This latter event is not affected in the *pex2* mutants; however, the two nuclei of the top dikaryotic cell do not fuse. Instead, they divide mitotically to produce another crozier cell (see arrow in Figure 4D). Consequently, mutant perithecia become filled with crozier “trees” (Figure 4C) in which no diploid stage can be detected (Simonet and Zickler, 1972, 1978; Berteaux-Lecellier et al., 1995).

In many fungi, karyogamy and meiosis are coupled processes. They are intimately associated to the cell differentiation process driving asci formation. Therefore, peroxisomes could be required either for karyogamy or meiosis *per se*, or they could be implicated in determining a differentiated cellular state competent to trigger karyogamy and meiosis.

The haploid nuclei of vegetative hyphae in *P. anserina* occasionally fuse and produce diploid nuclei. The frequency of this vegetative nuclear fusion is not affected upon *pex2* mutation, suggesting that the nuclear fusion *per se* is not impaired by this peroxisomal dysfunction (Berteaux-Lecellier et al., 1995). In filamentous Ascomycetes, the pre-meiotic DNA replication precedes karyogamy (reviewed in Zickler, 2006) and meiosis-specific proteins are incorporated into chromosomes during this phase (Storlazzi et al., 2008). Thus, the “decision” to undergo meiosis is taken before karyogamy in these fungi. Therefore, peroxisomes may be implicated in a prekaryogamy process necessary to induce meiosis rather than in karyogamy itself.

#### Does meiotic development require an alternative peroxisome import pathway?

In *P. anserina*, PEX2 localizes to peroxisomes during vegetative and sexual cycles, including crozier cells (Peraza-Reyes et al., 2008, 2011). Furthermore, meiocyte differentiation is also impaired by deletion of either *pex3* or *pex19*, which encode the peroxins implicated in peroxisome membrane assembly (Peraza-Reyes et al., 2011). This indicates that peroxisomes themselves are required for meiocyte formation. PEX2 and the entire RING-finger complex are required for meiocyte differentiation (Peraza-Reyes et al., 2008). Intriguingly, neither of the receptors PEX5 and PEX7 is required for karyogamy or meiosis initiation, and even double mutants lacking both import receptors can differentiate meiocytes and undergo karyogamy and meiosis (Bonnet et al., 2006). Further research revealed that the *P. anserina* docking-complex peroxin PEX13 is also required for meiocyte formation, whereas PEX14 (Figure 4B) and PEX14/17 are not (Peraza-Reyes et al., 2008, 2011).

These puzzling results suggested an unconventional/additional import pathway operating in *P. anserina* independently of the known import receptors. This hypothesis was further strengthened by the discovery that the PTS2-coreceptor PEX20 (Figure 4D) is essential for meiocyte formation (Peraza-Reyes et al., 2011). This finding, along with the notion that PTS2-correceptors display important functional similarities with PEX5 (Schafer et al., 2004; Schliebs and Kunau, 2006; Hensel et al., 2011), suggests that PEX20 could act as import receptor on its own (see Figure 3). Altogether, these data strongly suggest that meiocyte formation in *P. anserina* relies on a novel peroxisome import pathway, mediated by PEX20 and dependent on the docking protein PEX13 and the RING-finger complex.

The precise mechanism by which matrix proteins are imported into peroxisomes is not well-understood. Most notably, in contrast to the PTS1 import pathway, very little is known about the protein translocation channel for the PTS2 pathway. Nevertheless, based on their functional similarity with Pex5, the PTS2-coreceptors are appealing candidates for pore-forming activity (Schafer et al., 2004; Erdmann and Schliebs, 2005). It is, therefore, tempting to speculate that PEX20 provides a second pore-forming activity in filamentous Ascomycetes. This would indicate that *P. anserina* has distinct translocons for proteins that contribute to different stages of sexual development.

Interestingly, a genome-wide analysis of meiotic factors in *S. cerevisiae* revealed a requirement for the PTS2-correceptor Pex21p in meiotic development (Marston et al., 2004). In addition, Pex2p, Pex12p, and Pex13p (importomer peroxins), Pex6p and Pex22p (exportomer peroxins), and Pex25p (implicated in peroxisome division and biogenesis from the ER) were also identified as required for meiotic development. In contrast, deletion of the import receptors Pex5p and Pex7p, as well as the second PTS2-correceptor, Pex18p (a Pex21p paralog), was not observed to affect meiosis in this screen (Marston et al., 2004). These data could indicate a specialization for meiotic development for one of the two PTS2-coreceptors of *S. cerevisiae*. So far, no clear ortholog of *PEX20* has been observed in basidiomycete fungi (Kiel et al., 2006 and our personal observations); however, these fungi possess a Pex5/Pex20 fusion protein (Kiel et al., 2006), which could provide a separate pathway for peroxisome import. Whether this protein is implicated in sexual development in basidiomycete fungi remains undetermined.

The peroxisomal function required to induce meiosis in *P. anserina* remains elusive. The fatty acid β-oxidation pathway (either peroxisomal or mitochondrial) is not required for meiocyte formation (Boisnard et al., 2009). Likewise, simultaneous deletion of the five catalase-encoding genes in *P. anserina* does not impair meiocyte differentiation (Bourdais et al., 2012), which suggests that this process is not severely affected by deregulating the ROS homeostasis. The elimination of the peroxins that impair meiocyte formation in *P. anserina* results in different meiotic development defects in *S. cerevisiae* (Marston et al., 2004), and does not affect ascospore formation in *P. pastoris* (Waterham et al., 1996). Therefore, peroxisomes could participate in a specific function required for the differentiation of croziers/asci in filamentous fungi. However, the meiotic development of *A. nidulans* is not affected upon deletion of Pex2 or Pex13 (Hynes et al., 2008, 2010). This indicates that the involvement of peroxisomes in meiotic initiation is restricted to specific fungi, and suggests that the factors controlling meiotic entry among diverse fungi are different. Since the mating system of the homothallic *A. nidulans* differs from that of heterothallic *P. anserina*, one such difference could be related to the mating-type that governs sexual reproduction.

#### Involvement of peroxisomes in the distribution of meiotic-derived nuclei

In addition to meiotic initiation, progression through meiotic/post-meiotic development also requires peroxisome activity. In the homothallic *G. zeae*, the elimination of the import receptor Pex5 or the exportomer component Pex6 compromises asci and ascospore differentiation. The perithecia that are produced by mutant strains defective for these peroxins frequently lack asci and do not develop mature ascospores (Min et al., 2012). In *P. anserina*, the number of asci with well-delineated ascospores present in perithecia is also reduced by elimination of PEX5. In this fungus, delayed meiosis and nuclear misplacement during post-meiotic mitosis results in many degenerated asci, in which ascospores contain either no nuclei or an abnormal number of nuclei. These nuclear distribution abnormalities could result from incorrect spindle positioning during the second meiotic division or from impaired nuclear migration during post-meiotic mitosis (Bonnet et al., 2006). In *P. anserina*, elimination of the import receptor PEX7 and elimination of PEX5 have similar effects on asci development, although defects in *pex7* mutants are less severe compared to those observed in *pex5* mutants. In addition, the nuclear distribution defect of *pex5* mutants also displays a maternal effect (Bonnet et al., 2006). This indicates that the defect in nuclear positioning during asci development is not due to a cell autonomous effect, and suggests that maternally derived cells facilitate efficient progression through meiosis/post-meiotic development in a peroxisome-dependent manner.

In *A. nidulans*, microtubule-cytoskeleton and nuclear dynamics depend on the protein ApsB. This protein is a component of microtubule-organizing centers at the SPBs and at a septum-associated microtubule-organizing center, which is specific to fungal cells. In addition, ApsB localizes to a subpopulation of peroxisomes and it has been postulated that peroxisomes may deliver ApsB to its septal localization (Zekert et al., 2010). Thus, peroxisomes could have additional roles in regulating nuclear distribution during the sexual cycle. Actually, elimination of ApsB in *A. nidulans* results in misshapen ascospores produced in low numbers and exhibiting very low viability (Clutterbuck, 1994). Nevertheless, this phenotype has not been observed in any of the analyzed peroxisome biogenesis deficient mutants (Hynes et al., 2008, 2010). Therefore, the contribution of the peroxisomal form of ApsB to ascospore differentiation could be minor, or redundant systems could ensure ApsB correct localization during sexual development. Further research to better understand the involvement of peroxisomes in cytoskeleton organization could reveal additional roles for these organelles in meiotic development.

### Involvement of peroxisomes in sexual sporulation

#### The glyoxylate cycle, sensing the metabolic potential to promote sexual spore formation

Interestingly, in *S. cerevisiae* the glyoxylate cycle is a central pathway linking the catabolic and biosynthetic metabolism, and also senses the metabolic potential of the cell to promote developmental decisions, like sexual spore formation. Sexual sporulation in *S. cerevisiae* is controlled by the nutritional status. Nitrogen starvation in media containing non-fermentable carbon sources, substrates like acetate that require glyoxylate bypass, induces sporulation (Neiman, 2011). In addition, progression through sporulation is under the control of the nutritional status. For instance, if nutrients are exhausted once meiosis has initiated, two ascospores are produced inside each ascus instead of four.

Interestingly, the number of spores an ascus will form is determined by a metabolic product of the glyoxylate cycle (Nickas et al., 2004). Ascospore formation relies on the activity of the SPBs. At the second meiotic division, the composition of the SPBs changes and they are converted from microtubule nucleation centers into membrane nucleation platforms (referred to as meiosis-II outer plaques) that direct the formation of the membranes that will surround each ascospore (Neiman, 2011). Upon carbon depletion, only one (the daughter) of the two SPBs of each spindle is transformed into a meiosis-II outer plaque, which results in the formation of two spores. Accumulation of a glyoxylate cycle-derived intermediate, which may be cytosolic oxaloacetate, triggers the modification of the two mother SPBs into meiosis-II outer plaques allowing the formation of four ascospores (Nickas et al., 2004). This indicates that the asymmetric spindle pole function is regulated by the metabolic state of the cell and that the glyoxylate cycle plays a major monitoring role in this process. This underscores the importance of the glyoxylate cycle beyond its metabolic function. Importantly, the localization of glyoxylate cycle enzymes in *S. cerevisiae* varies according to nutrient availability. For example, these enzymes localize primarily to peroxisomes during growth on fatty acids. In contrast, most of them localize to cytosol upon growth on C2 compounds. The oxaloacetate-consuming citrate synthase, which has mitochondrial and peroxisomal isoforms in yeast, is probably the only exception (Kunze et al., 2002, 2006; Lee et al., 2011; Chen et al., 2012). This indicates that different conformations of the glyoxylate cycle may be optimal to sustain different tasks, and suggests that the location where the intermediates of this pathway are produced could contribute to different cellular roles.

#### Peroxisome metabolism is required for sexual spore maturation and germination

Research on *P. anserina* sexual development revealed that peroxisome number increases importantly during ascospore maturation (Berteaux-Lecellier et al., 1995). A similar observation was done from asexual spores of *G. zeae* (Seong et al., 2008). Interestingly, the abundance of peroxisomes in the asexual spores of this fungus is correlated with a high number of lipids droplets in their vicinity, which disappear during germination. These observations suggest that peroxisomes are involved in spore maturation and germination, where they could drive the mobilization of the spore reserve lipids.

Ascospores of *P. anserina* peroxisomal mutants are green colored instead of black (Figure 4, compare A and B) display a low germination rate and a flimsy mycelium after germination (Berteaux-Lecellier et al., 1995; Bonnet et al., 2006; Peraza-Reyes et al., 2008; Boisnard et al., 2009). Melanin constitutes an important component of fungal sexual spores (Gomez and Nosanchuk, 2003). This pigment can be produced by the dihydroxynaphthalene melanin biosynthesis pathway from either acetyl-CoA or malonyl-CoA (Langfelder et al., 2003; Ramos-Pamplona and Naqvi, 2006; Coppin and Silar, 2007). Therefore, β-oxidation pathway could provide precursors for melanin biosynthesis. Melanization of ascospores in *P. anserina* (Berteaux-Lecellier et al., 1995; Bonnet et al., 2006; Peraza-Reyes et al., 2008) and appressoria in phytopathogenic fungi is affected by defects in peroxisome function (Kimura et al., 2001; Ramos-Pamplona and Naqvi, 2006). Indeed, melanization is also deficient in mutants defective for peroxisome β-oxidation pathway (Wang et al., 2007; Boisnard et al., 2009), indicating a role for this pathway in melanization. Importantly, a *P. anserina* mutant defective in both peroxisomal and mitochondrial β-oxidation pathways produces green ascospores, while a mutant defective for the first step of melanin biosynthesis gives rise to white ascospores (Coppin and Silar, 2007). Thus, β-oxidation contributes to, but it is not the only metabolic pathway providing precursors for melanin biosynthesis. Additional sources of melanin precursors could also explain why teliospores of *U. maydis mfe2* mutants, affected in peroxisome β-oxidation, only display delayed melanization (Klose and Kronstad, 2006). Interestingly, plant pathogenic fungi could obtain these biosynthetic precursors from the infected plants (Guenther et al., 2009).

Considering that melanin helps to harden the spore cell wall, the less-pigmented ascospores of *P. anserina* peroxisomal mutants should be fragilized. Indeed, deeper studies have shown that the germination defect of these ascospores is caused by their increased fragility (Boisnard et al., 2009). This is in line with the observation that *A. nidulans* peroxisomal mutant ascospores, for which no melanization defect has been reported, as well as the teliospores of *U. maydis mfe2* mutant, which only harbor a delayed melanization, germinate efficiently (Klose and Kronstad, 2006; Hynes et al., 2008).

Germination of *P. anserina* peroxisomal mutant ascospores gives rise to a spindly mycelium with reduced growth rate (Berteaux-Lecellier et al., 1995; Bonnet et al., 2006; Peraza-Reyes et al., 2008; Boisnard et al., 2009). This phenotype disappears when glucose is added to the germination medium (Berteaux-Lecellier et al., 1995). Similarly, glucose addition suppresses the germination defect of *Aspergillus fumigatus* asexual spores defective for isocitrate lyase or malate synthase (Olivas et al., 2008). This suggests that when external resources are limiting, growth of germinative mycelia in these fungi can be sustained by spore reserve compounds, whose mobilization requires peroxisome activity.

In *N. crassa*, absence of isocitrate lyase compromises ascospore germination, which underscores the importance of glyoxylate cycle in this process. Furthermore, in this fungus addition of sucrose or TCA cycle intermediates does not improve ascospore germination of isocitrate lyase mutants (Flavell and Fincham, 1968). Interestingly, triacylglycerides constitute a major metabolic resource for germinating ascospores in *N. crassa* (Goodrich-Tanrikulu et al., 1998), whereas they represent only a minor proportion of the asexual spore lipids (Bianchi and Turian, 1967). Consistently, isocitrate lyase-deficient asexual spores of *N. crassa* do not exhibit germination defects (Flavell and Fincham, 1968).

Transfer of β-oxidation-derived acetyl-CoA into mitochondria for energy generation may be important for ascospore germination. However, elimination of CATs has an ambiguous impact on ascospore germination in different fungi. While ascospore germination is slightly reduced in *S. sclerotiorum pth2* null strains (Liberti et al., 2013), deletion of its ortholog exerts no developmental defect in *A. nidulans* (Hynes et al., 2011), and results in precocious germination in *G. zeae* (Son et al., 2012). Furthermore, defective glyoxylate cycle or peroxisome assembly does not affect sexual or asexual spore viability in *A. nidulans* (Armitt et al., 1976; Gainey et al., 1992; Hynes et al., 2008). Altogether, these data indicate that requirement for peroxisomes during spore germination depends on the constitution and metabolic resources of a spore, which can importantly vary between different fungi and depending on the sexual or asexual origin of spores.

Research on the role of peroxisomes in the sexual spores of fungal lineages that are not ascomycetes is scant. However, the presence of malate synthase activity in basidiospores (the meiotic-derived sexual spores of Basidiomycetes) of different lineages of Agaricomycetes—the mushroom-forming fungi—suggests a widespread occurrence of the glyoxylate cycle is these spores (Ruch et al., 1991). Furthermore, similarly to asexual spores of *G. zeae*, cytological and ultrastructural analyses have revealed lipid stores in basidiospores, which are resolved as numerous lipid droplets in close proximity to microbodies that are probably peroxisomes and mitochondria (Ruch and Motta, 1987; Ruch et al., 1991). These observations suggest that the fatty acid β-oxidation and the glyoxylate cycle could also be important for lipid reserve mobilization during the germination of basidiomycete sexual spores.

#### A role for peroxisomes in forcibly ascospore discharge

Research in *G. zeae* uncovered an additional involvement for peroxisomes in asci development, which is not related to meiotic development or ascospore formation *per se* (Son et al., 2012). After meiosis completion and ascospore formation, the remaining original ascus cell consists of a sac-like structure, which encases the ascospores. In many filamentous ascomycetes, tubular asci are perforated at their tips upon spore maturation and then act as “water cannons,” which forcibly expel their ascospores out of the ascus (for review, Trail, 2007). In *G. zeae* mutants lacking the peroxisomal/mitochondrial carnitine acetyltransferase (CAT1) the maturation of asci and ascospores is normal; however, ascospore discharge is considerably reduced. This phenotype is aggravated by deletion of the gene encoding a second carnitine acetyltransferase (CAT2), which localizes to cytosol and peroxisomes (Son et al., 2012).

Ascospore ejection relies on the turgor pressure generated inside asci after an increase in its osmolyte concentration drives an influx of water (Trail, 2007). In the CAT mutants, ascospores remain clustered in *cirri* at the perithecium ostiole (see Figure 1) instead of been effectively expelled out from perithecia (Son et al., 2012). This suggests a deficient turgor pressure generation inside asci. The precise function of CATs in this process remains elusive. However, since turgor pressure generation probably consumes high levels of ATP (Son et al., 2012), the defect in ascospore ejection in CAT mutants could reflect the need for acetyl-CoA, which is required for mitochondrial ATP production. Defects in ascospore ejection have also been observed in *P. anserina* strains deficient for β-oxidation in mitochondria or for the PTS2 receptor PEX7. However, the ejection of ascospores in these mutants appears to be only delayed (Bonnet et al., 2006; Boisnard et al., 2009). These observations indicate that, in addition to their formation and germination, peroxisomes also participate in the dispersal of sexual spores.

## Concluding remarks

Fungi represent a large group of organisms, which exhibit high diversity in terms of lifestyles and reproductive strategies. Peroxisomes contribute to this diversity by providing metabolic versatility, which allows fungi to colonize a broad range of environments. The functional versatility provided by peroxisomes extends to the orchestration of developmental processes, like sexual reproduction. Remarkably, peroxisomes have been directly or indirectly implicated in most major developmental events driving sexual reproduction in fungi, including the formation of sexual regulatory signaling molecules, mating, meiotic induction and progression, as well as the differentiation, dispersal and germination of sexual spores. Moreover, the developmental processes accompanying sexual reproduction, like the differentiation and sustenance of the sexual reproductive structures, also critically require the activity of peroxisomes. Interestingly, during these processes peroxisomes allow metabolic relocation not only between somatic cells and sexual reproductive structures, but also between different cell types within the sexual fructifications, and between different multicellular differentiated structures. However, peroxisome involvement in the sexual cycle varies among fungal species and little conservation is so far apparent for many peroxisome developmental functions. Although further comparative research is required to better appreciate the occurrence of such diverse developmental roles, these observations underscore the versatility of peroxisomes in fungi, which seem to be remarkably adaptable and capable of adopting different roles. They underline also the functional diversity of peroxisomes, which clearly plays an important role in the diversity of the developmental systems that have evolved in fungi over 1 billion years of evolution.

### Conflict of interest statement

The authors declare that the research was conducted in the absence of any commercial or financial relationships that could be construed as a potential conflict of interest.
